# A regulatory role for β-adrenergic receptors regarding the resolvin D1 (RvD1) pathway in the diabetic retina

**DOI:** 10.1371/journal.pone.0185383

**Published:** 2017-11-02

**Authors:** Haoshen Shi, Thomas W. Carion, Youde Jiang, Adam Chahine, Jena J. Steinle, Elizabeth A. Berger

**Affiliations:** 1 Department of Anatomy & Cell Biology, Wayne State University School of Medicine, Detroit, Michigan, United States of America; 2 Department of Ophthalmology, Kresge Eye Institute, Detroit, Michigan, United States of America; University of Florida, UNITED STATES

## Abstract

Diabetic retinopathy is a visually debilitating disease with limited treatment options available. Compound 49b, a β-adrenergic receptor agonist, has been demonstrated to effectively reduce disease pathogenesis associated with diabetic retinopathy. While the exact mechanisms are not fully understood, previous studies have determined that it reduces the pro-inflammatory cytokine, TNF-α, and inhibits apoptosis of the retinal microvasculture. As inflammation becomes more recognized in driving disease pathogenesis, so does the regulation by pro-resolving pathways as therapeutic points of intervention. The current study sought to explore whether Compound 49b had any influence on pro-resolving pathways, thus contributing to improved disease outcome. Using *in vivo* (animal model of type 1 diabetes) and *in vitro* (retinal endothelial cells, Müller cells, neutrophils/PMN) techniques, it was determined that high glucose lowers pro-resolving lipid mediator, resolvin D1 (RvD1) levels and differentially alters required enzymes, 5-lipoxygenase (5-LOX), 15-LOX-1 and 15-LOX-2. RvD1 receptors formyl peptide receptor 2 (ALX/FPR2) and G-protein coupled receptor 32 (GPR32) were also downregulated in response to hyperglycemic conditions. Moreover, it was observed that β-adrenergic receptor activation restored high glucose-induced decreases in both enzyme activity and RvD1 levels observed *in vivo* and *in vitro*. The current study is the first to describe a regulatory role for β-adrenergic receptors on pro-resolving pathways.

## Introduction

Roughly 10% of Americans were diagnosed with diabetes in 2012 (National Diabetes Statistics Report, 2014). Given that nearly 60% of diabetic patients will develop some complications related to diabetic retinopathy, generation of novel therapies is of paramount importance. While it is clear that the retinal response to high glucose is multifactorial, including oxidative stress, vascular endothelial cell growth factor (VEGF), protein kinase C, inflammatory mediators, endoplasmic reticulum stress, and epigenetic changes, therapies to prevent or delay progression of diabetic retinopathy continue to elude scientists. It has been previously reported that docosahexaenoic acid (DHA) is abundant in the retina [1]. This omega-3 fatty acid has recently been linked to the activation of a number of enzymes and proteins reported to be anti-inflammatory, as well as protective to tissues [2, 3]. Among them includes resolvin D1 (RvD1), a D-series resolvin [4, 5], which has been shown to display potent anti-inflammatory activity and control the inflammation-resolution balance in host defense. Hypoxia was one of the early signals found to stimulate the production of pro-resolving mediators, such as RvD1 and RvE1, as demonstrated in hypoxic endothelial cells [4]. Work in the oxygen-induced retinopathy (OIR) model of proliferative retinopathy has suggested that RvD1 is protective within the retina, which was correlated with reduced TNF-α levels [6]. Furthermore, 4-hydroxy-docosahexaenoic acid (4-HDHA), an intermediate metabolite of DHA through 5-lipoxygenase (5-LOX) activity, also reduced retinal neovascularization [7]. This response was subsequently blocked when 5-LOX was eliminated [7].

In order to generate RvD1, DHA is metabolized by both 5- and 15-LOX [4] [5], which selectively interacts with receptors ALX/FPR2 and GPR32 [8]. Using the streptozotocin model of type 1 diabetes, it was found that loss of 5-LOX (a key lipid mediator enzyme) resulted in reduced vascular damage, oxidative stress, and leukostasis [9]. Work in *db/db* mice has revealed that local application of RvD1 accelerated wound closure and decreased apoptotic cell accumulation [10]. Further studies using *db/db* mice revealed that RvD1 treatment was protective against development of type 2 diabetes [11]. Additionally, studies in HepG2 cells demonstrated that RvD1 attenuated endoplasmic reticulum stress-induced apoptosis in a model of non-alcoholic fatty liver disease [12]. Taken together, data from multiple tissues suggest that the pathological markers associated with diabetes lead to a significant reduction in resolvins, which may further contribute to exacerbated retinal damage.

We have previously reported that Compound 49b, a β-adrenergic receptor agonist, demonstrates both anti-apoptotic and anti-inflammatory properties in the diabetic retina and in retinal cells under hyperglycemic conditions [13]. For that reason, we questioned whether β-adrenergic receptor signaling may regulate lipoxygenase enzyme expression and resultant RvD1 production, particularly in two resident retinal cell types, REC and Müller cells. Polymorphonuclear leukocytes (PMN) were examined as well, in light of increased leukostasis and the mounting pathogenic role of inflammatory cells during the development of diabetic retinopathy. There is little on the role of β-adrenergic receptor regulation of lipid mediators in the eye. *In vitro* studies have demonstrated that protein kinase A (PKA) can phosphorylate 5-LOX in PMN [14]. In contrast, work in human airway endothelial cells suggests that 15-LOX-1 can decrease β2-adrenergic receptor phosphorylation, leading to decreased cAMP levels [15]. Additionally, mouse models of Alzheimer’s disease have shown that norepinephrine induces expression of formyl peptide receptor 2 (ALX/FPR2) [16], which is one of two known receptors for RvD1 [17, 18]. Thus, the potential regulatory role for β-adrenergic receptor signaling on RvD1 or lipoxygenase enzymes in a diabetic retinopathy model remains unknown.

We have previously reported that Compound 49b can reduce TNF-α, as well as SOCS3, under hyperglycemic conditions [13, 19]. Therefore, we hypothesized that diabetes or high glucose culturing conditions would decrease enzymatic levels of 15-LOX and downstream production of RvD1, which could be ameliorated by Compound 49b. Indeed, we found that high glucose and diabetic conditions significantly decreased 15-LOX, as well as RvD1 levels. In addition, lipoxygenase enzymes and RvD1 were increased following Compound 49b treatment.

## Experimental procedures

### Animals

All mouse experiments were approved by the Institutional Animal Care and Use Committee at Wayne State University (Protocol# 11-08-14). C57BL/6J wildtype mice were purchased from Charles River Laboratories. Mice were made diabetic by injections of 60 mg/kg of streptozotocin dissolved in citrate buffer for 5 consecutive days. Control mice received citrate buffer only. Glucose measurements were done weekly, with glucose levels >250 mg/dL considered diabetic. At 2 months of diabetes, 10 control and 10 diabetic mice received Compound 49b (4 μL containing 1 mM) (formulated by Dr. Duane Miller, University of Tennessee Health Science Center, Memphis TN, in collaboration with Dr. Jena Steinle) topically onto each eye for 14 days. After 14 days of Compound 49b treatment, all mice were sacrificed and analyzed as described below.

### Retinal endothelial cell culture

Primary human retinal microvascular endothelial cells (REC) were acquired from Cell System Corporation (CSC, Kirkland, Washington). Cells were grown in M131 medium containing microvascular growth supplements (Invitrogen), 10 μg/mL gentamycin, and 0.25 μg/mL amphotericin B. Prior to the experiment, cells were transferred to high (25 mM) or normal (5 mM) glucose medium (M131 medium with added glucose), supplemented with MVGS and antibiotics for 3 days. Only primary cells within passage 6 were used. Cells were quiesced by incubating in high or normal glucose medium without MVGS for 24 hr. Compound 49b treatment was then added at 50 nM for 24 hours, as done previously [13].

### Müller cell (rMC-1) culture

Müller cells (rMC-1; kindly provided by Dr. Vijay Sarthy at Northwestern University) were thawed and cultured in DMEM medium under normal glucose (5mM) conditions. Medium was supplemented with 10% FBS and antibiotics. Once cells reached ~ 80% confluency, they were passed into dishes containing either high (25 mM) or normal glucose medium. Once ready for experimentation, cells were moved to the appropriate medium without FBS to induce serum starvation for 18–24 hours. Compound 49b was then applied at 50 nM for 24 hours prior to cell collection.

### PMN isolation

Peritoneal PMN from C57BL/6 mice were harvested as previously described [20]. In brief, mice received an intraperitoneal (IP) injection (1.0 mL) of a 9% casein solution (Difco, Detroit, MI) administered 27 hours prior to cell harvest, followed by a second injection 24 hours later. Cells were collected by peritoneal lavage 3 hours after the second injection, washed 3× (200 ×*g*, 10 min), and then isolated using a Percoll gradient (100,000 ×*g*, 20 min). Cell viability (>95%) and purity (>90%) were determined. Cells were resuspended in media (RPMI 1640 supplemented with 3% FCS and antibiotics) containing either normal glucose (5 mM) or high glucose (25 mM) for 24 hours (37⁰C, 5% CO_2_). Cells were then exposed to Compound 49b treatment (50 nM) for an additional 24 hours, prior to harvest for protein analyses.

### Cell treatment

REC were selectively treated with either propranolol, a β-adrenergic receptor antagonist, or PKA siRNA (Dharmacon, Lafayette, CO). For propranolol work, cells were cultured under normal and high glucose conditions, and treated with propranolol (50 nM) 30 minutes prior to 49b treatment. To block the PKA pathway, cells were similarly cultured and transfected with PKA siRNA at a final concentration of 20 nM as previously described [21] using GenMute siRNA transfection kit (SignaGen, Rockville, MD) followed by 49b treatment.

### Western blotting

After appropriate treatments and rinsing with cold phosphate-buffered saline, REC, rMC-1 and PMN were collected in lysis buffer containing protease and phosphatase inhibitors and scraped into tubes. Retinal extracts were prepared by sonication. Equal amounts of protein from the cell or tissue extracts were separated on pre-cast tris-glycine gels (Invitrogen, Carlsbad, CA), and then blotted onto nitrocellulose membranes. After blocking in TBST (10mM Tris-HCl buffer, pH 8.0, 150 mM NaCl, 0.1% Tween 20) and 5% (w/v) BSA, membranes were treated with the following primary antibodies: 5-LOX, 15-LOX-1, 15-LOX-2, ALX/FPR2, GPR32 (Abcam, San Francisco, CA) and β-actin (Santa Cruz, Santa Cruz, CA), followed by incubation with appropriated secondary antibodies (Fisher Scientific, Pittsburgh, PA) labeled with horseradish peroxidase. Antigen-antibody complexes were detected using a chemilluminescent reagent kit (Thermo Scientific, Pittsburgh, PA). Western blot images were collected on an Azure Biosystem C500 machine (Azure Biosystems, Dublin, CA) and densitometric analysis was performed.

### ELISA

RvD1 ELISA kits (Cayman Chemical, Ann Arbor, MI) were used to measure RvD1 expression in REC, rMC-1, PMN and retinal lysates. Equal protein concentrations were added to all wells. Assay protocol was performed according to the manufacturer’s instructions. Cross reactivity of this assay for RvD2 and RvE1 is 0.05% and <0.01%, respectively.

### Statistics

All experiments were repeated in triplicate and data are presented as the mean ± SEM. Non-parametric Kruskal-Wallis with Dunn’s post-hoc tests were used for the cell culture data. One-way ANOVA with a Student Newman Keul’s post-hoc test was done for animal studies using Prism 7.0 software. *P*<0.05 was considered significant. Representative blots are shown for all Western blot analyses.

## Results

### Compound 49b significantly increases RvD1 levels and receptors ALX/FPR2 and GPR32 in the diabetic retina

**Fig 1:**
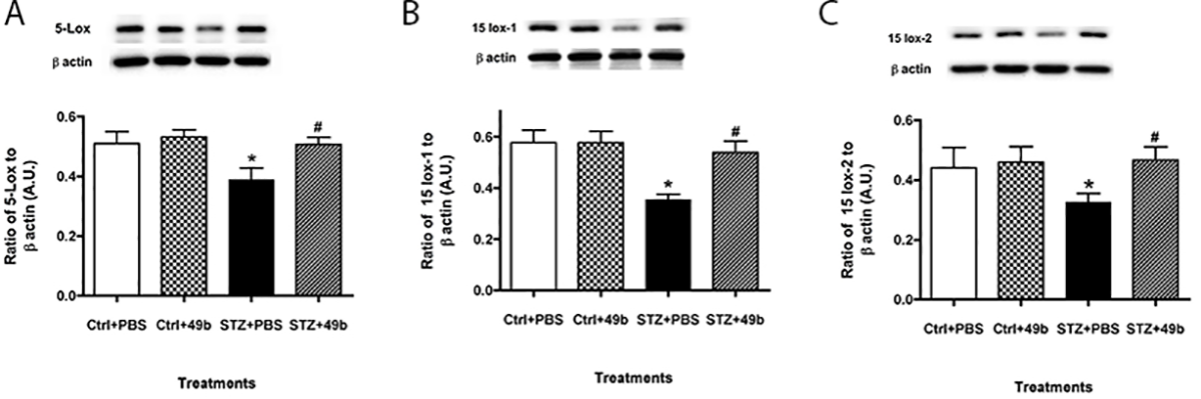
Compound 49b significantly increased RvD1 (A), ALX/FPR2 (B) and GPR32 (C) in the diabetic retina. Data from whole retinal lysates from C57BL/6 mice untreated (Ctrl+PBS), control mice treated with Compound 49b (Ctrl+49b), streptozotocin-induced diabetic mice (STZ+PBS), or diabetic mice treated with Compound 49b (STZ+49b). *P<0.05 vs. ctrl+PBS, ^#^P<0.05 vs. STZ+PBS. N = 5 mice in each group.

Since DHA is highly abundant in the retina [1], we determined whether RvD1 levels and associated receptors, ALX/FPR2 and GPR32 [17, 18] were altered under diabetic conditions (Fig 1A–1C). In 2-month diabetic mice, levels of RvD1 (A), ALX/FPR2 (B) and GPR32 (C) were significantly decreased. In contrast, Compound 49b treatment significantly enhanced expression of all three proteins when compared to untreated diabetic mice. These results suggest that Compound 49b may be protective to the diabetic retina through key pro-resolving pathways.

### Compound 49b prevents high glucose-induced decrease in RvD1 and associated receptors in REC

**Fig 2:**
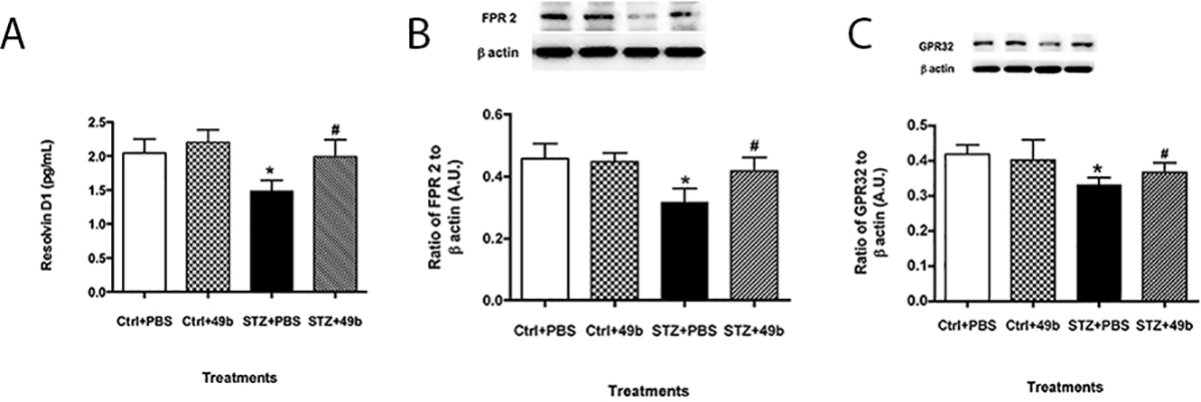
Compound 49b increased levels of RvD1 (A), ALX/FPR2 (B) and GPR32 (C) in REC exposed to high glucose. REC cells were grown in normal glucose (NG), normal glucose+Compound 49b (NG+49b), high glucose (HG) or high glucose treated with Compound 49b (HG+49b). *P<0.05 vs. NG, ^#^P<0.05 vs. HG. N = 4 for each treatment.

To determine which cell types may contribute to the observed RvD1 profile in the diabetic retina, REC in normal and high glucose were treated with Compound 49b. Parallel to the diabetic retina, RvD1 levels were significantly decreased in REC exposed to high glucose conditions (Fig 2A). RvD1 receptors ALX/FPR2 and GPR32 were similarly downregulated with high glucose (Fig 2B and 2C, respectively). After treatment with Compound 49b, RvD1, ALX/FPR2, and GPR32 levels were significantly upregulated in REC, despite hyperglycemic conditions, to levels similarly observed with normal glucose.

### Compound 49b results in differential expression of lipoxygenase enzymes in REC

**Fig 3:**
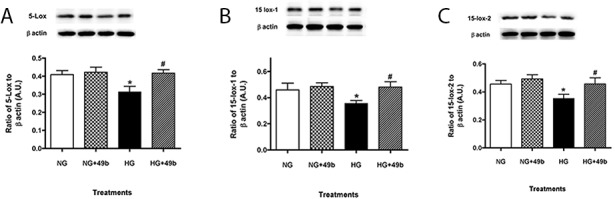
Lipoxygenase enzymes were differentially expressed in REC cells grown in high glucose and after Compound 49b treatment. 5-LOX (A), 15-LOX-1 (B) and 15-LOX-2 (C) in REC cells grown in normal glucose (NG), normal glucose+Compound 49b (NG+49b), high glucose (HG) or high glucose treated with Compound 49b (HG+49b). *P<0.05 vs. NG, ^#^P<0.05 vs. HG. N = 4 for each treatment.

5-LOX, 15-LOX-1 and 15-LOX-2 enzymes were significantly increased in REC cultured in high glucose (Fig 3A–3C). When REC grown in high glucose were treated with Compound 49b, 5-LOX enzyme expression returned to levels observed in normal glucose (Fig 3A), with further enhanced expression of 15-LOX-1 and 15-LOX-2 (Fig 3B and 3C), suggesting that β-adrenergic receptor signaling may differentially regulate key lipid mediator enzymes in REC.

### Compound 49b did not affect RvD1, ALX/FPR2 or GPR32 in Müller cells

**Fig 4:**
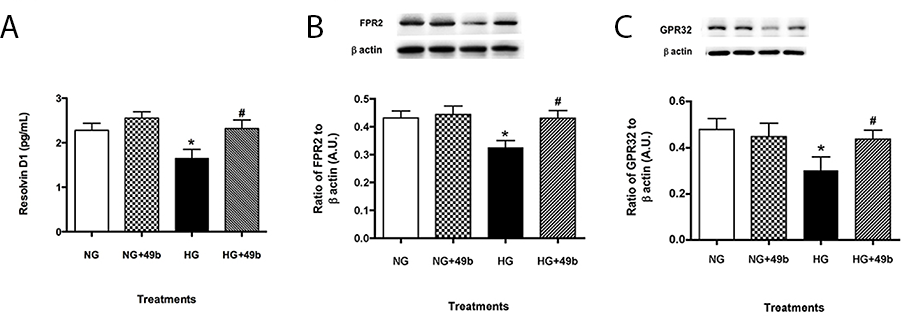
Müller cells grown in high glucose did not increase RvD1 levels after Compound 49b treatment. Müller cells were grown in in normal glucose (NG), normal glucose+Compound 49b (NG+49b), high glucose (HG) or high glucose treated with Compound 49b (HG+49b). Panel A shows RvD1 protein expression, panels B and C show Western blot results for ALX/FPR2, and GPR32 levels in Müller cells. *P<0.05 vs. NG, ^#^P<0.05 vs. HG. N = 4 for each treatment.

Since Müller cells are the primary residential inflammatory cell type in the retina, we examined whether they respond likewise to high glucose via changes in the D-series resolvin pathway. As illustrated in Fig 4, neither high glucose nor Compound 49b altered RvD1 levels in Müller cells (A). Similar responses were displayed regarding ALX/FPR2 and GPR32 when compared to REC and whole retinal lysates–levels were decreased in Müller cells in response to high glucose (Fig 4B and 4C). However, Compound 49b had no apparent effect.

### Compound 49b exhibited limited effects on high glucose-induced changes in lipoxygenase enzymes in Müller cells

**Fig 5:**
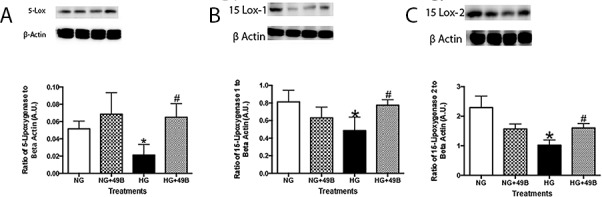
Compound 49b displayed limited effects on 5-LOX in Müller cells in high glucose. Müller cells were grown in in normal glucose (NG), normal glucose+Compound 49b (NG+49b), high glucose (HG) or high glucose treated with Compound 49b (HG+49b). Panels A–C illustrate Western blots for 5-LOX, 15-LOX-1 and 15-LOX-2, respectively. *P<0.05 vs. NG, ^#^P<0.05 vs. HG. N = 4 for each treatment.

As shown in Fig 5, 5-LOX (A) was significantly upregulated, while 15-LOX-1 enzyme (B) was downregulated in Müller cells grown in high glucose; no differences were observed with 15-LOX-2 (C). Compound 49b effectively reduced 5-LOX levels to those similar to control. However, unlike REC and the diabetic retina, Compound 49b treatment had no effect on either 15-LOX expression in Müller cells cultured in high glucose.

### High glucose conditions decreased RvD1, ALX/FPR2 and GPR32 levels in PMN

**Fig 6:**
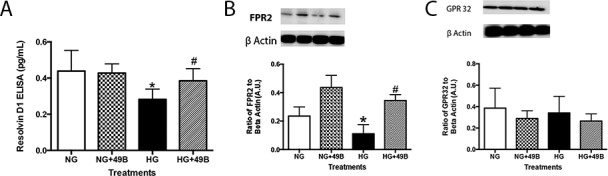
Compound 49b significantly increased ALX/FPR2 levels, with no influence on RvD1 or GPR32 levels. RvD1 (A), ALX/FPR2 (B) and GPR32 (C) expression in PMN exposed to normal glucose (NG), normal glucose+Compound 49b (NG+49b), high glucose (HG) or high glucose treated with Compound 49b (HG+49b). *P<0.05 vs. NG, ^#^P<0.05 vs. HG. N = 4 for each treatment.

To investigate a systemic immune cell type that may respond to retinal damage, isolated murine PMN were exposed to normal and high glucose +/- Compound 49b treatment. Protein levels of RvD1, ALX/FPR2 and GPR32 were detected in PMN (Fig 6A–6C). Exposure to high glucose resulted in significant downregulation of all three molecules. Compound 49b did not appear to affect RvD1 or GPR32 –levels remained unchanged under both normal glucose and high glucose conditions. However, hyperglycemia-induced decreases in ALX/FPR2 were restored after Compound 49b treatment.

### Compound 49b had limited effect on lipoxygenase levels in PMN in high glucose

**Fig 7:**
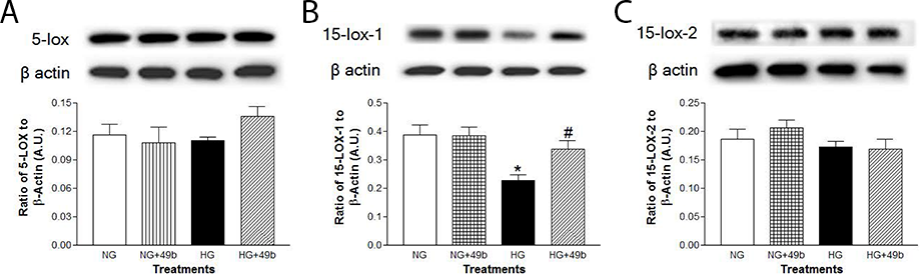
Compound 49b had no effect on lipoxygenase levels in PMN exposed to high glucose. Mouse PMN cells were exposed to normal glucose (NG), normal glucose+Compound 49b (NG+49b), high glucose (HG) or high glucose treated with Compound 49b (HG+49b). Western blots results are shown for 5-LOX (A), 15-LOX-1 (B) and 15-LOX-2 (C) expression. *P<0.05 vs. NG, ^#^P<0.05 vs. HG. N = 4 for each treatment.

High glucose conditions significantly reduced 15-LOX-1 levels in PMN, with no effect observed with 5-LOX or 15-LOX-2 (Fig 7A–7C). Following Compound 49b treatment, levels for all three enzymes high glucose remained unchanged. These results suggest that systemic immune cells may respond differently to β-adrenergic receptor agents compared to residential retinal cells.

### Compound 49b effects on RvD1 in REC are dependent on β-adrenergic receptor signaling

**Fig 8:**
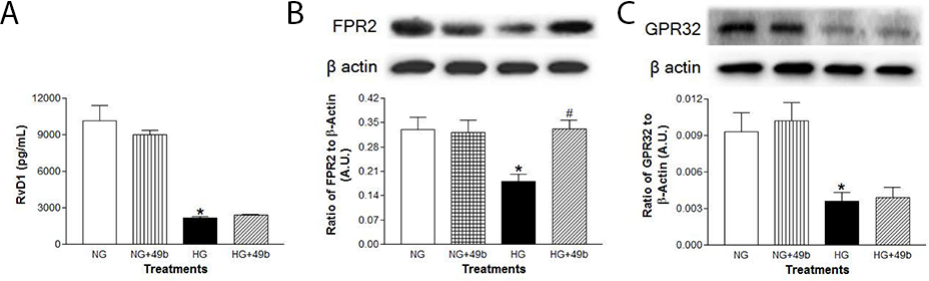
Increased RvD1 levels after Compound 49b treatment in REC were β-adrenergic receptor pathway specific. REC were exposed to normal glucose (NG), normal glucose+Compound 49b (NG+49b), normal glucose+Compound 49b+propranolol or PKA siRNA(NG+49b+propranolol/siPKA), high glucose (HG), high glucose treated with Compound 49b (HG+49b) and high glucose+Compound 49b+propranolol/PKA siRNA (HG+49b+propranolol/siPKA). ELISA results are shown for RvD1 levels after treatment with propranolol (A) and PKA siRNA (B). *P<0.05 vs. NG, ^#^P<0.05 vs. HG. N = 4 for each treatment.

Compound 49b activity is carried out through β-adrenergic receptor mediated increases in PKA activity [13]. To determine the specificity of Compound 49b’s observed influence on the pro-resolving RvD1 pathway, REC were exposed to propranolol, a non-specific β-adrenergic receptor antagonist, or PKA siRNA in the presence of high glucose and/or Compound 49b. As shown in Fig 8A, propranolol abrogated the effects of Compound 49b and resulted in RvD1 levels similar to those observed with high glucose alone. Similar effects were seen with PKA siRNA (Fig 8B), whereby RvD1 levels were significantly reduced comparable to high glucose alone despite Compound 49b treatment, thus further suggesting a β-adrenergic receptor signaling pathway-dependent effect.

## Discussion

Work in the OIR model has suggested that lipid mediators may reduce retinal neovascularization, with lipoxygenase enzymes expressed primarily by circulating cells [7]. Additionally, it has been demonstrated that RvD1 and RvE1, as well as neuroprotectin, are protective in the OIR model, potentially through reduced TNF-α levels [6]. It is well established that early diabetic retinopathy following streptozotocin (STZ) injections leads to increased TNF-α levels [13, 22, 23]. Work in diabetic-induced 5-LOX knockout mice demonstrated that loss of this key enzyme resulted in reduced degenerate capillaries, leukostasis, and NF-κB levels, while reduced leukostasis only was observed in diabetic 12/15-LOX knockout mice [9]. Furthermore, work in humans has suggested that patients with diabetic retinopathy or diabetic macular edema have decreased levels of resolvins and protectins [24]. Thus, it appears that diabetes (both proliferative and non-proliferative) may alter lipoxygenase levels, as well as resolvin activity. However, the regulation and cellular source of these enzymes and resolving pathways is less clear. Regarding the former, there are limited and conflicting data regarding the potential regulatory role for β-adrenergic receptor signaling in relation to RvD1 or lipoxygenase enzymes [14–16]. Based upon these studies, it is likely that different cell types and/or systems could potentially activate different signal transduction pathways for resolvin production specific to each system. Hence, the current study sought to define potential regulatory pathways associated with RvD1 expression in the diabetic retina and begin to identify the cellular source(s) related to these pathways.

Compound 49b is a β-adrenergic receptor agonist based structurally on isoproterenol with chemical modifications to increase its ocular potency for use as a topical treatment [13]. It has been previously shown to significantly reduce cleaved caspase 3 in the diabetic retina and retinal endothelial cells [13, 25]. Moreover, we have demonstrated that hyperglycemia-induced increases in TNF-α in REC and Müller cells [19, 26] can be effectively reduced by Compound 49b [19, 26]. It has also been found that the protective effect of RvD1 against diabetic retinopathy occurs in part through the suppression of TNF-α [6]. Therefore, we determined whether Compound 49b could regulate lipoxygenase enzymes and resultant RvD1 expression, ultimately contributing to the reduction in TNF-α both *in vitro* (REC and Müller cell culture) and *in vivo* (STZ-induced diabetic retina). We also examined PMN to extend our findings to circulating inflammatory cells, given the role of leukostasis during development and progression of diabetic retinopathy. Overall, our data indicate that high glucose significantly reduces RvD1 and corresponding receptor levels in the diabetic retina. Our findings in the STZ-induced diabetic mouse retina are in contrast to findings in the OIR model, where RvD1 levels were not altered [7]. The observed differences in RvD1 could be due to glucose-induced damage versus oxygen-induced damage. However, ALX/FPR2 and GPR32 (RvD1 receptors) were also decreased in the diabetic retina. In addition, similar results were consistently observed for these same molecules tested in REC after exposure to high glucose conditions. Correspondingly, treatment with Compound 49b significantly increased RvD1 expression similar to normal glucose both in the diabetic retina and *in vitro* using REC accompanied by decreased 5-lipoxygenase and increased 15-lipoxygenase enzyme levels.

Upon further examination of retinal Müller cells, a slightly different response was observed. While high glucose resulted in increased 5-LOX and decreased 15-LOX, ALX/FPR2 and GPR32 levels, RvD1 remained unchanged. This suggests that Müller cells have a different profile in response to high glucose than the whole retina or REC, with no change in RvD1 levels, but downregulated receptor expression. Similar to REC and diabetic retina; however, Compound 49b significantly reduced 5-LOX enzyme levels in Müller cells cultured in high glucose, as well as increased ALX/FPR2 levels, suggesting a potential regulatory pathway in Müller cells, albeit much less responsive. Compound 49b did not affect GPR32 levels, indicating that RvD1 would most likely signal through ALX/FPR2 in Müller cells and warrants further investigation. In the diabetic retina, REC and Müller cells, β-adrenergic receptor signaling appears to be pro-resolving, at least in part, through actions carried out by RvD1 and ALX/FPR2. However, this needs to be further confirmed using ALX/FPR2 antagonists and/or silencing of the PI3K/Akt signaling pathway to determine the extent of RvD1/ALX/FPR2 in mediating the protective effects of Compound 49b. Further, given the increased 5-LOX enzyme levels, it does not rule out the potential role Müller cells may have in producing 5-lipoxygenase-driven pro-inflammatory lipid mediators, such as leukotrienes.

When we expanded our work to circulating inflammatory cells (namely PMN) that are known to enter the retina during leukostasis, yet a different paradigm appeared. Effects of high glucose culturing conditions resulted in decreased RvD1, ALX/FPR2 and GPR32 levels; though were limited to decreased 15-LOX-1 regarding lipoxygenase enzymes. Despite no response of 15-LOX-2 to high glucose exposure, these results are not surprising given that 15-LOX-1 is highly expressed by circulating leukocytes compared to 15-LOX-2, which is thought to be more restricted to tissue expression. And though 5-LOX (and 15-LOX-2) remain unchanged under high glucose conditions, these results suggest that 15-LOX-1 may be the limiting enzyme in the production of RvD1 within the PMN. Furthermore, while RvD1, 15-LOX-1, ALX/FPR2 and GPR32 were significantly decreased in high glucose, only ALX/FPR2 was restored toward normal glucose levels after Compound 49b treatment. Albeit a limited receptor effect, it may have significant implications as RvD1 has been shown to potently regulate both human and mouse neutrophils [4] [5].

These results suggest that although retinal cells appear to lose pro-resolving and lipoxygenase enzyme responses in high glucose, restoration through stimulation of β-adrenergic receptor signaling pathways may occur. As such, we next confirmed that the observed effects of Compound 49b in RECs were carried out through activation of β-adrenergic receptor signaling, as indicated using propranolol–a non-specific β-adrenergic receptor antagonist. In addition, this specificity was further confirmed using PKA siRNA to silence the downstream β-adrenergic receptor signaling pathway, as both approaches resulted in blunted RvD1 expression under high glucose conditions despite Compound 49b treatment. In contrast, while circulating PMN are partially responsive to high glucose, they did not appear to be influenced by the β-adrenergic receptor agonist. This raises two points as to why PMN alone are only partially responsive to high glucose compared to the response of retinal cells. One potential explanation for the limited response to high glucose may be related to the stimuli. PMN are designed to circulate throughout the body and respond to specific damage. The 25mM glucose used in this study may not be an appropriate damage stimulus for PMN. Likewise, it is possible that other pro-inflammatory signals released from retinal cells or cell-cell contact with REC may be required to elicit a more robust response from neutrophils. To this end, the microenvironment of the retinal vasculature may allow for interplay between REC and PMN that can be influenced by both high glucose conditions and Compound 49b treatment.

Another key question arising from this study is why β-adrenergic receptors may regulate RvD1? In the retina, it is clear that maintenance of β-adrenergic receptor signaling reduces inflammatory pathways [13, 27–29]. Although it has been reported in activated microglia that isoproterenol (from which Compound 49b is derived) increases (as opposed to decreases) levels of inflammatory cytokines [30] [31], studies were carried out in chronic stress and surgical trauma models–not models of high glucose or hyperglycemia. To this end, we have shown a different effect of 49b on REC and Müller cells. Compound 49b works through β1-adrenergic receptors in REC and β2-adrenergic receptors. In both cases of these cell types, Compound 49b reduces TNF-α [13][32]. Once we established the actions on TNF-α, we then moved to show its actions on TLR4. From these findings, we hypothesized it may work on pro-resolving pathways, as well. To date, little to no information exists on β-adrenergic receptors and RvD1. Our findings that Compound 49b can increase RvD1 in diabetic whole retina and REC grown in high glucose strongly indicate that this may occur through β-adrenergic actions on cAMP. Work in rat brain astrocytes demonstrated that DHA release can be regulated by calcium-independent phospholipase A2 (iPLA2) [33]. DHA release was amplified by PKA agonist application [33]. Since we have shown that Compound 49b significantly increases PKA [13], it is possible that our findings on lipoxygenase enzymes and RvD1 may relate to actions on PKA and iPLA2. These will be the focus of further study.

Collectively, these data indicate for the first time a regulatory role for β-adrenergic receptors regarding pro-resolving pathways in the diabetic retina. Activation of β-adrenergic receptor signaling pathway via Compound 49b rescued hyperglycemic-induced decreases in RvD1, its key enzymes 5- and 15-LOX, as well as receptors ALX/FPR2 and GPR32 in the diabetic retina and REC. Future insight into how β-adrenergic receptor signaling pathways influence pro-resolving mediators in the diabetic retina may contribute to the development of therapeutic modalities targeted at the resolution of inflammation, not simply anti-inflammatory in nature.

## References

[1] Lydic TA, Renis R, Busik JV, Reid GE. Analysis of Retina and Erythrocyte Glycerophospholipid Alterations in a Rat Model of Type 1 Diabetes. JALA Charlottesv Va. 2009;14(6):383–99. doi: 10.1016/j.jala.2009.07.003 ; PubMed Central PMCID: PMCPMC2786180. 20161420 10.1016/j.jala.2009.07.003 PMC2786180

[2] Opreanu M, Lydic TA, Reid GE, McSorley KM, Esselman WJ, Busik JV. Inhibition of cytokine signaling in human retinal endothelial cells through downregulation of sphingomyelinases by docosahexaenoic acid. Investigative ophthalmology & visual science. 2010;51(6):3253–63. doi: 10.1167/iovs.09-4731 ; PubMed Central PMCID: PMCPMC2891477. 20071681 10.1167/iovs.09-4731 PMC2891477

[3] Chen W, Esselman WJ, Jump DB, Busik JV. Anti-inflammatory effect of docosahexaenoic acid on cytokine-induced adhesion molecule expression in human retinal vascular endothelial cells. Investigative ophthalmology & visual science. 2005;46(11):4342–7. doi: 10.1167/iovs.05-0601 ; PubMed Central PMCID: PMCPMC1378111. 16249517 10.1167/iovs.05-0601 PMC1378111

[4] Serhan CN, Hong S, Gronert K, Colgan SP, Devchand PR, Mirick G, et al Resolvins: a family of bioactive products of omega-3 fatty acid transformation circuits initiated by aspirin treatment that counter proinflammation signals. J Exp Med. 2002;196(8):1025–37. doi: 10.1084/jem.20020760 ; PubMed Central PMCID: PMCPMC2194036. 12391014 10.1084/jem.20020760 PMC2194036

[5] Sun YP, Oh SF, Uddin J, Yang R, Gotlinger K, Campbell E, et al Resolvin D1 and its aspirin-triggered 17R epimer. Stereochemical assignments, anti-inflammatory properties, and enzymatic inactivation. J Biol Chem. 2007;282(13):9323–34. doi: 10.1074/jbc.M609212200 . 17244615 10.1074/jbc.M609212200

[6] Connor KM, SanGiovanni JP, Lofqvist C, Aderman CM, Chen J, Higuchi A, et al Increased dietary intake of omega-3-polyunsaturated fatty acids reduces pathological retinal angiogenesis. Nat Med. 2007;13(7):868–73. doi: 10.1038/nm1591 17589522 10.1038/nm1591 PMC4491412

[7] Sapieha P, Stahl A, Chen J, Seaward MR, Willett KL, Krah NM, et al 5-Lipoxygenase metabolite 4-HDHA is a mediator of the antiangiogenic effect of omega-3 polyunsaturated fatty acids. Sci Transl Med. 2011;3(69):69ra12 doi: 10.1126/scitranslmed.3001571 ; PubMed Central PMCID: PMCPMC3711031. 21307302 10.1126/scitranslmed.3001571 PMC3711031

[8] Krishnamoorthy S, Recchiuti A, Chiang N, Fredman G, Serhan CN. Resolvin D1 receptor stereoselectivity and regulation of inflammation and proresolving microRNAs. Am J Pathol. 2012;180(5):2018–27. doi: 10.1016/j.ajpath.2012.01.028 ; PubMed Central PMCID: PMCPMC3349829. 22449948 10.1016/j.ajpath.2012.01.028 PMC3349829

[9] Gubitosi-Klug RA, Talahalli R, Du Y, Nadler JL, Kern TS. 5-Lipoxygenase, but not 12/15-lipoxygenase, contributes to degeneration of retinal capillaries in a mouse model of diabetic retinopathy. Diabetes. 2008;57(5):1387–93. doi: 10.2337/db07-1217 ; PubMed Central PMCID: PMCPMC4444435. 18346986 10.2337/db07-1217 PMC4444435

[10] Tang Y, Zhang MJ, Hellmann J, Kosuri M, Bhatnagar A, Spite M. Proresolution therapy for the treatment of delayed healing of diabetic wounds. Diabetes. 2013;62(2):618–27. doi: 10.2337/db12-0684 ; PubMed Central PMCID: PMCPMC3554373. 23043160 10.2337/db12-0684 PMC3554373

[11] Hellmann J, Tang Y, Kosuri M, Bhatnagar A, Spite M. Resolvin D1 decreases adipose tissue macrophage accumulation and improves insulin sensitivity in obese-diabetic mice. FASEB journal: official publication of the Federation of American Societies for Experimental Biology. 2011;25(7):2399–407. doi: 10.1096/fj.10-178657 ; PubMed Central PMCID: PMCPMC3114534. 21478260 10.1096/fj.10-178657 PMC3114534

[12] Jung TW, Hwang HJ, Hong HC, Choi HY, Yoo HJ, Baik SH, et al Resolvin D1 reduces ER stress-induced apoptosis and triglyceride accumulation through JNK pathway in HepG2 cells. Molecular and cellular endocrinology. 2014;391(1–2):30–40. doi: 10.1016/j.mce.2014.04.012 . 24784707 10.1016/j.mce.2014.04.012

[13] Zhang Q, Guy K, Pagadala J, Jiang Y, Walker RJ, Liu L, et al Compound 49b Prevents Diabetes-Induced Apoptosis through Increased IGFBP-3 Levels. Investigative ophthalmology & visual science. 2012;53(6):3004–13. Epub 2012/04/03. doi: 10.1167/iovs.11-8779 22467575 10.1167/iovs.11-8779 PMC3378083

[14] Werz O, Szellas D, Steinhilber D, Radmark O. Arachidonic acid promotes phosphorylation of 5-lipoxygenase at Ser-271 by MAPK-activated protein kinase 2 (MK2). The Journal of biological chemistry. 2002;277(17):14793–800. doi: 10.1074/jbc.M111945200 . 11844797 10.1074/jbc.M111945200

[15] Albano GD, Zhao J, Etling EB, Park SY, Hu H, Trudeau JB, et al IL-13 desensitizes beta2-adrenergic receptors in human airway epithelial cells through a 15-lipoxygenase/G protein receptor kinase 2 mechanism. The Journal of allergy and clinical immunology. 2015;135(5):1144–53 e1-9. doi: 10.1016/j.jaci.2015.02.006 ; PubMed Central PMCID: PMCPMC4426258. 25819984 10.1016/j.jaci.2015.02.006 PMC4426258

[16] Kong Y, Ruan L, Qian L, Liu X, Le Y. Norepinephrine promotes microglia to uptake and degrade amyloid beta peptide through upregulation of mouse formyl peptide receptor 2 and induction of insulin-degrading enzyme. J Neurosci. 2010;30(35):11848–57. doi: 10.1523/JNEUROSCI.2985-10.2010 . 20810904 10.1523/JNEUROSCI.2985-10.2010 PMC6633413

[17] Krishnamoorthy S, Recchiuti A, Chiang N, Yacoubian S, Lee CH, Yang R, et al Resolvin D1 binds human phagocytes with evidence for proresolving receptors. Proceedings of the National Academy of Sciences of the United States of America. 2010;107(4):1660–5. doi: 10.1073/pnas.0907342107 ; PubMed Central PMCID: PMCPMC2824371. 20080636 10.1073/pnas.0907342107 PMC2824371

[18] Norling LV, Dalli J, Flower RJ, Serhan CN, Perretti M. Resolvin D1 limits polymorphonuclear leukocyte recruitment to inflammatory loci: receptor-dependent actions. Arterioscler Thromb Vasc Biol. 2012;32(8):1970–8. doi: 10.1161/ATVBAHA.112.249508 ; PubMed Central PMCID: PMCPMC3401489. 22499990 10.1161/ATVBAHA.112.249508 PMC3401489

[19] Walker RJ, Anderson NM, Jiang Y, Bahouth S, Steinle JJ. Role of beta-adrenergic receptors regulation of TNF-alpha and insulin signaling in retinal Muller cells. Investigative ophthalmology & visual science. 2011;52(13):9527–33. Epub 2011/11/24. doi: 10.1167/iovs.11-8631 22110065 10.1167/iovs.11-8631 PMC3341119

[20] Berger EA, McClellan SA, Vistisen KS, Hazlett LD. HIF-1alpha is essential for effective PMN bacterial killing, antimicrobial peptide production and apoptosis in Pseudomonas aeruginosa keratitis. PLoS Pathog. 2013;9(7):e1003457 doi: 10.1371/journal.ppat.1003457 ; PubMed Central PMCID: PMCPMC3715414. 23874197 10.1371/journal.ppat.1003457 PMC3715414

[21] Walker RJ, Steinle JJ. Role of beta-adrenergic receptors in inflammatory marker expression in Muller cells. Invest Ophthalmol Vis Sci. 2007;48(11):5276–81. doi: 10.1167/iovs.07-0129 . 17962483 10.1167/iovs.07-0129

[22] Joussen AM, Doehmen S, Le ML, Koizumi K, Radetzky S, Krohne TU, et al TNF-alpha mediated apoptosis plays an important role in the development of early diabetic retinopathy and long-term histopathological alterations. Molecular vision. 2009;15:1418–28. 9014. 19641635 PMC2716944

[23] Kern TS. Contributions of inflammatory processes to the development of the early stages of diabetic retinopathy. Exp Diabetes Res. 2007;2007:95103 Epub 2008/02/16. doi: 10.1155/2007/95103 PubMed Central PMCID: PMC2216058. 18274606 10.1155/2007/95103 PMC2216058

[24] Das UN. Lipoxins, resolvins, and protectins in the prevention and treatment of diabetic macular edema and retinopathy. Nutrition. 2013;29(1):1–7. doi: 10.1016/j.nut.2012.02.003 . 22677359 10.1016/j.nut.2012.02.003

[25] Zhang Q, Jiang Y, Miller MJ, Peng B, Liu L, Soderland C, et al IGFBP-3 and TNF-alpha Regulate Retinal Endothelial Cell Apoptosis. Investigative ophthalmology & visual science. 2013;54(8):5376–84. doi: 10.1167/iovs.13-12497 PubMed Central PMCID: PMC3741024. 23868984 10.1167/iovs.13-12497 PMC3741024

[26] Zhang Q, Steinle JJ. IGFBP-3 inhibits TNF-alpha production and TNFR-2 signaling to protect against Retinal Endothelial Cell Apoptosis. Microvascular research. 2014;95:76–81. doi: 10.1016/j.mvr.2014.07.009 25086184 10.1016/j.mvr.2014.07.009 PMC4346193

[27] Walker R, Steinle J. Role of Beta-adrenergic Receptors in Inflammatory Marker Expression in Muller Cells. Investigative ophthalmology & visual science. 2007;48(11):5276–81. 8753. 17962483 10.1167/iovs.07-0129

[28] Panjala SR, Jiang Y, Kern TS, Thomas SA, Steinle JJ. Increased tumor necrosis factor-alpha, cleaved caspase 3 levels and insulin receptor substrate-1 phosphorylation in the beta-adrenergic receptor knockout mouse. Molecular vision. 2011;17:1822–8. Epub 2011/08/19. 9369; PubMed Central PMCID: PMC3137556. 21850156 PMC3137556

[29] Steinle JJ. Sympathetic neurotransmission modulates expression of inflammatory markers in the rat retina. Experimental eye research. 2007;84(1):118–25. doi: 10.1016/j.exer.2006.09.006 17067575 10.1016/j.exer.2006.09.006

[30] Johnson JD, Zimomra ZR, Stewart LT. Beta-adrenergic receptor activation primes microglia cytokine production. J Neuroimmunol. 2013;254(1–2):161–4. doi: 10.1016/j.jneuroim.2012.08.007 . 22944319 10.1016/j.jneuroim.2012.08.007

[31] Wang J, Li J, Sheng X, Zhao H, Cao XD, Wang YQ, et al Beta-adrenoceptor mediated surgery-induced production of pro-inflammatory cytokines in rat microglia cells. J Neuroimmunol. 2010;223(1–2):77–83. doi: 10.1016/j.jneuroim.2010.04.006 . 20452680 10.1016/j.jneuroim.2010.04.006

[32] Jiang Y, Zhang Q, Ye EA, Steinle JJ. beta1-adrenergic receptor stimulation by agonist Compound 49b restores insulin receptor signal transduction in vivo. Mol Vis. 2014;20:872–80. ; PubMed Central PMCID: PMCPMC4067233. 24966659 PMC4067233

[33] Strokin M, Sergeeva M, Reiser G. Docosahexaenoic acid and arachidonic acid release in rat brain astrocytes is mediated by two separate isoforms of phospholipase A2 and is differently regulated by cyclic AMP and Ca2+. British journal of pharmacology. 2003;139(5):1014–22. doi: 10.1038/sj.bjp.0705326 ; PubMed Central PMCID: PMCPMC1573920. 12839876 10.1038/sj.bjp.0705326 PMC1573920

